# Supplementary material for: Selective Intra-Arterial Doxorubicin Eluting Microsphere Embolization for Desmoid Fibromatosis: A Combined Prospective and Retrospective Study
Source: Cancers (Basel). 2022 Oct 14;14(20):5045. doi: 10.3390/cancers14205045 (PMC9599870; doi:10.3390/cancers14205045)
Supplement: Supplementary file 1 [file cancers-14-05045-s001.zip › cancers-1892754-supplementary.pdf]

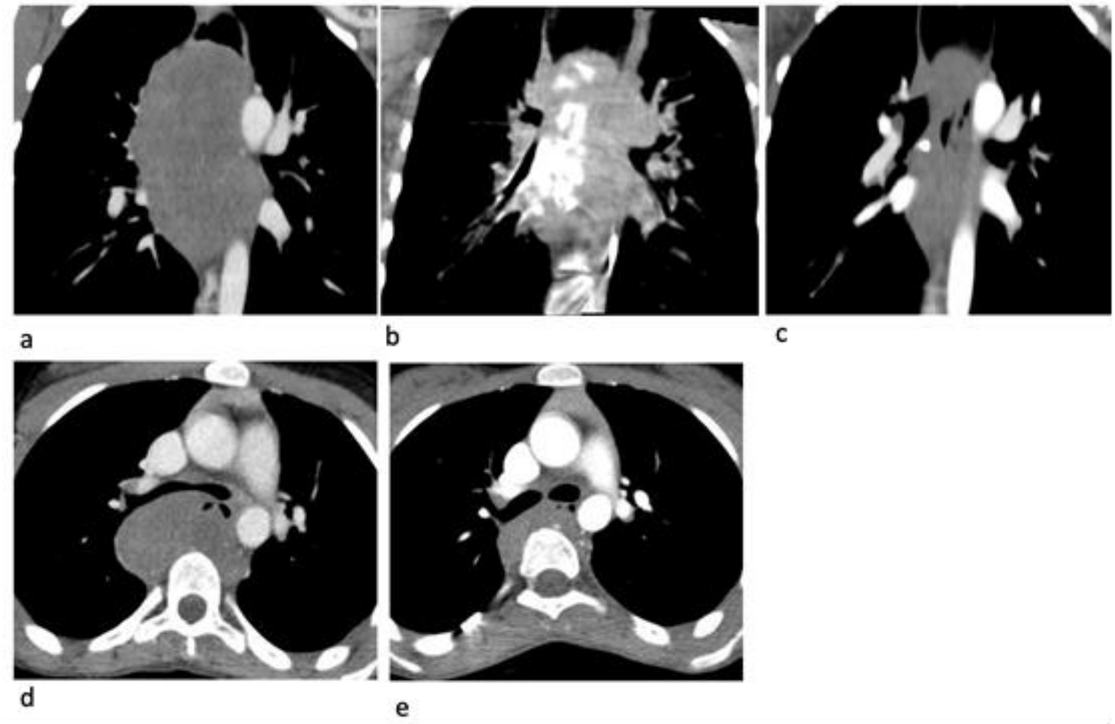


**Figure S1.** Baseline coronal (a) and axial (b) images of a posterior mediastinal DFs causing obliteration of the esophageal lumen and airway compression in a 13-year-old girl. Cone beam CT demonstrates contrast opacifying the tumor from a selective injection at the right bronchial artery (c). Six-month follow-up coronal (d) and axial (e) imaging demonstrate decreased mass effect upon the airways following DEE. The DFs volume decrease was 65%, whereas the longest axis decrease (cranial–caudal) was only 10%. The patient had stable disease by RECIST criteria.
